# How climate, landscape, and economic changes increase the exposure of *Echinococcus* Spp.

**DOI:** 10.1186/s12889-022-14803-4

**Published:** 2022-12-10

**Authors:** Xiaoyu Di, Shuo Li, Bin Ma, Xiaofan Di, Yuhao Li, Bei An, Wenwen Jiang

**Affiliations:** 1 Department of Pathogenic Biology, School of Basic Medical Sciences, Lanzhou University, Lanzhou, China; 2 The Second Hospital of Lanzhou University, Lanzhou, China; 3 Laboratory Medicine Center, The Second Hospital of Lanzhou University, Lanzhou, China

**Keywords:** MaxEnt model, Gansu Province, Climate change, Global shared socio-economic pathways (SSPs) scenarios, Echinococcosis, Ecotourism

## Abstract

**Background:**

Echinococcosis is a global enzootic disease influenced by different biological and environmental factors and causes a heavy financial burden on sick families and governments. Currently, government subsidies for the treatment of patients with echinococcosis are only a fixed number despite patients’ finical income or cost of treatment, and health authorities are demanded to supply an annual summary of only endemic data. The risk to people in urban areas or non-endemic is increasing with climate, landscape, and lifestyle changes.

**Methods:**

We conducted retrospective descriptive research on inpatients with human echinococcosis (HE) in Lanzhou hospitals and analyzed the healthcare expenditure on inpatient treatment and examined the financial inequalities relating to different levels of gross domestic product. The livestock losses were also estimated by infection ratio. The occurrence records of *Echinococcus* spp. composed of hospitalized patients and dogs infected in the Gansu province were collected for Ecological niche modeling (ENM) to estimate the current suitable spatial distribution for the parasite in Gansu province. Then, we imported the resulting current niche model into future global Shared Socioeconomic Pathways scenarios for estimation of future suitable habitat areas.

**Results:**

Between 2000 to 2020, 625 hospitalized HE patients (51% men and 49% women) were identified, and 48.32 ± 15.62 years old. The average cost of hospitalization expenses per case of HE in Gansu Province was ￥24,370.2 with an increasing trend during the study period and was negative with different counties’ corresponding gross domestic product (GDP). The trend of livestock losses was similar to the average cost of hospitalization expenses from 2015 to 2017. The three factors with the strongest correlation to echinococcosis infection probability were (1) global land cover (GLC, 56.6%), (2) annual precipitation (Bio12, 21.2%), and (3) mean temperature of the Wettest Quarter (Bio12, 8.5% of variations). We obtained a robust model that provides detail on the distribution of suitable areas for *Echinococcus* spp. including areas that have not been reported for the parasite. An increasing tendency was observed in the highly suitable areas of *Echinococcus* spp. indicating that environmental changes would affect the distributions.

**Conclusion:**

This study may help in the development of policies for at-risk populations in geographically defined areas and monitor improvements in HE control strategies by allowing targeted allocation of resources, including spatial analyses of expenditure and the identification of non-endemic areas or risk for these parasites, and a better comprehension of the role of the environment in clarifying the transmission dynamics of *Echinococcus* spp. Raising healthcare workers’ and travelers’ disease awareness and preventive health habits is an urgent agenda. Due to unpredictable future land cover types, prediction of the future with only climatic variables involved needs to be treated cautiously.

**Supplementary Information:**

The online version contains supplementary material available at 10.1186/s12889-022-14803-4.

## Background

Echinococcosis is a chronic parasitic infection of humans, domestic animals, and wildlife as a neglected parasitic disease [1], which gives precedent support for its elimination and control [2]. Treatment of human echinococcosis requires significant expenditure for the families of patients and countries [3]. Echinococcosis is usually expensive and causes a heavy financial burden on sick families [4]. Above 90% of the global burden of alveolar echinococcosis (AE) [5] and 34% of cystic echinococcosis (CE) are in China [6]. Currently, treatments in public hospitals in China are financed through three sources: government subsidies, patient fees, and drug markups. As poverty and infectious diseases reinforce each other, the Chinese government has committed to fighting and eliminating poverty-impoverished areas and has promoted health literacy for echinococcosis according to Healthy China 2030 [7]. The prevalence of human echinococcosis (HE) was positively related to the ethic ratio but negatively related to the Gross Domestic Product (GDP) [8]. Due to the high disease burden still shown in spite of national programme, appropriate policies and actions are demanded [3]. Echinococcosis is mostly distributed in endemic pastoral and semi-pastoral and poverty-stricken counties but few in non-endemic areas [6]. Health authorities are demanded to supply only an annual summary of endemic data. However, the risk to people non-endemic is increasing with climate, landscape, and lifestyle changes [4]. In the meantime, most endemic counties of echinococcosis are well-known tourist areas that receive thousands of visitors (local and outside) involved in ecotourism [9]. With ecotourism blooming [9], travelers are increasingly exposed to CE and AE by accidentally ingesting *Echinococcus* eggs in contaminated food, water, or soil [10].

Echinococcosis occurrence is highly dependent climatic factors including temperature and humidity [11–13] due to their influences on the survival of eggs and environment factors such as landscape factors (land use, grassland area ratio, vegetation coverage, land cover changes) [8]. Domestic dogs are the most important definitive host of both *E. granulosus* and E. multilocularis with the highest risk of transmitting CE and AE to humans due to their ability to wander freely in pastoral areas and prey on slaughtered live-stock. Landscape of the world has been altered, and novel environments have infiltrated [7, 10]. Due to anthropogenic land use, lifestyle changes, and even microclimate changes, afforestation [14], particularly farming and fencing practices are linked to the distribution and dynamics of *Echinococcus* spp. intermediate hosts [15]. Hence, study demands to be applied to better understand the ecological processes that may result in variations in the transmission patterns of *Echinococcus* spp. based on shifting environmental factors [16].

Environmental niche models (ENMs) are powerful tools that can enhance the insight of the potential and actual distribution of species in question, as well as the way in which environmental factors may influence a species [17]. Furthermore, these models can be applied to forecast the influences of motivations of global environmental change, such as climatic or landcover alterations on the distribution of parasites [16, 18]. Hence, integrating information about pure and projected environmental variations might allow us to prioritize inspecting activities to exploit the possibility of expecting the advent of pathogens, specifical mediators of endemic, emergent, and re-emergent zoonotic diseases in novel areas [18] .

Gansu Province is a co-endemic AE and CE areas with various environmental conditions belonging to the most impoverished region in China [19]. The temperature and precipitation in Gansu Province decrease from southeast to northwest, and the Hexi areas show an upward trend in annual precipitation [20]. There were 56 out of 368 Chinese endemic echinococcosis counties but uneven distributed in 81 counties and districts in Gansu Province [21]. Consequently, most investigations currently focus on endemic echinococcosis counties [21, 22]. However, some HE cases have been reported in Lanzhou City [23, 24]. Hence, it is very key to increase urban citizens and tourists’ awareness of risky practices that transmit the disease [25]. To our knowledge, no scientific studies have been conducted to analyze the health care expenditure on inpatient treatment and examined the financial inequalities relating to different levels of gross domestic product, determine their impacts [26] of climate and environmental changes on the shift in the distributional range of *Echinococcus* spp. in Gansu Province.

Here, we modeled the spatiotemporal dynamic distribution of *Echinococcus* spp. in Gansu Province from 2000 to 2020 by collecting echinococcosis scientific literature and hospital records, specifically quantifying impatient case characteristics. We aimed to address the following research questions: (1) describe patients’ characteristics related to echinococcosis at a province level to get a better picture of the current epidemiological scenario after the implementation of regional control programs, (2) estimate dynamics of *Echinococcus* spp. impact in terms of monetary losses including animal production losses and hospitalization expenditure in Gansu Province, (3) estimate the current spatial distribution of suitable conditions for the *Echinococcus* spp. in Gansu Province, (4) evaluate the potential influence of climatic change on the distribution of suitable conditions *Echinococcus spp* in the future (2070).

## Methods

### Study area

**Fig. 1:**
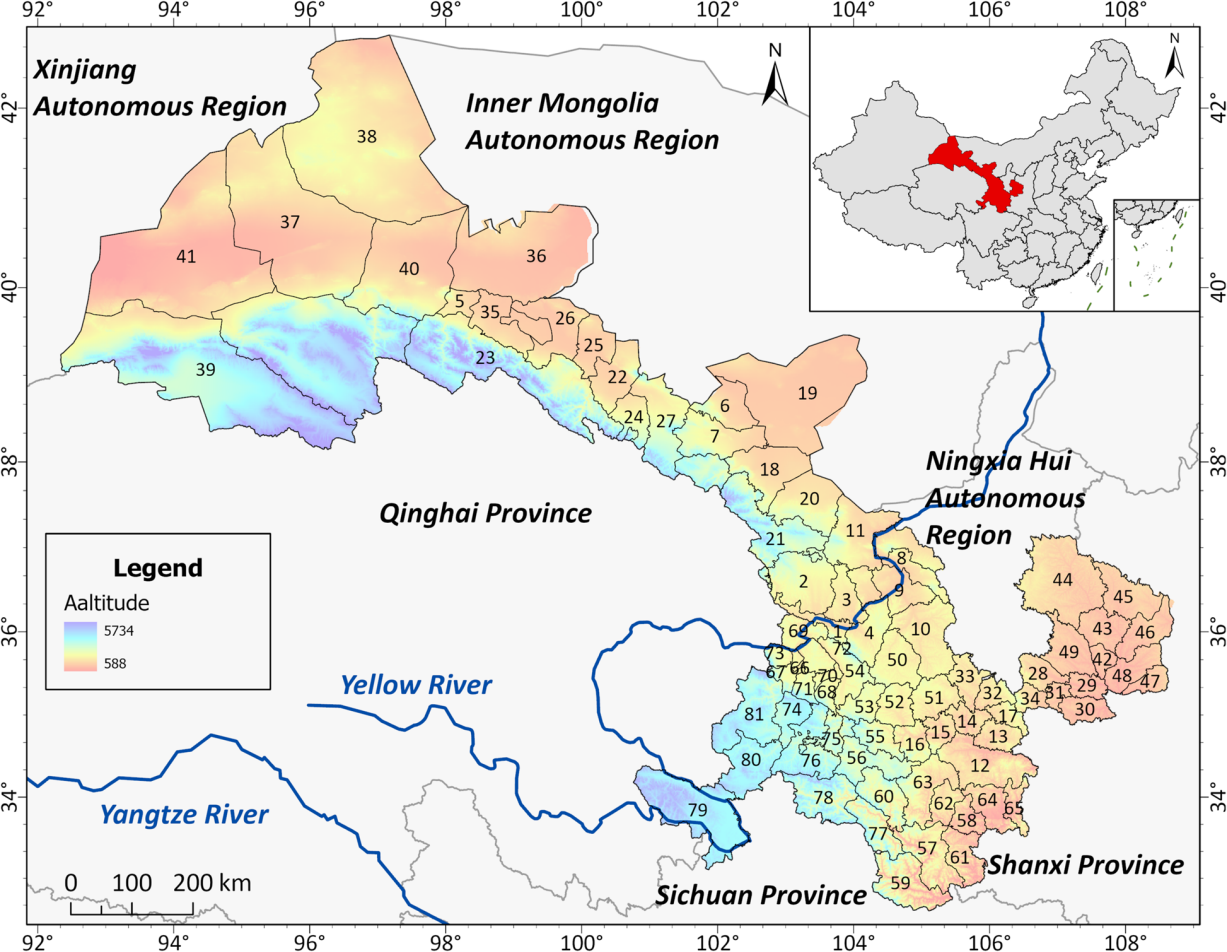
The geographical location of Gansu Province, China (Available online for the map layer of Altitude and Remote Sensing Image: www. Gscloud.cn/). numbers indicate different counties or districts (See Additional file 1 supple Excel A1). Note Map was created with ArcMap from ArcGIS 10.8 by our team

Gansu Province (32°110′N-42°570′N, 92°130′E-108°460′E) is situated at the intersection of the monsoon region in eastern China and the semiarid region in northwestern China (Fig. 1 including 81 counties and districts), bordering six endemic echinococcosis provinces [21].

### Collection of clinical information and livestock losses

We conducted a retrospective descriptive study of HE cases between 2000 to 2020 from three hospitals in Lanzhou. Sociodemographic (sex, age, and county of residency) and clinical characteristics (length of hospitalization, noninvasive procedures and history of surgical intervention during hospitalization, and hospitalization cost to the health care system, and financing regime) were collected. The length of hospitalization was calculated by applying the admission and discharge dates for each case. This research estimated the economic losses for 523 patients who underwent surgery or non-surgery and 103 patients were removed due to lack of hospital expenditure information. The cost involved hospitalization only covered medical fees (e.g., drugs, diagnosis-related exams, and surgery). We also conducted a retrospective descriptive study of livestock infected ratio to estimate animal productivity losses.

### Sampling collections and other occurrence data from public databases

To collect a database on the *Echinococcus* spp. sampling and detection localities, we carried out a systematic search in the Web of Science and China National Knowledge Infrastructure (CNKI) using the search terms “*Echinococcus*” or “*echinococcosis*” and “Gansu”, Distributions of dog and HE were collected through a literature review and national reports. Hence, the occurrence records of *Echinococcus* spp. composed of hospitalized patients and dogs infected in the Gansu province were collected for Ecological niche modeling (ENM) to estimate the current suitable spatial distribution for the parasite in Gansu province. When the available data lacked geographic coordinates, Google Earth 7.3.3 (https://about.google/) was applied to obtain the approximate longitude and latitude based on the depicted geographic sites. Species occurrence data were organized into “.csv” format files following workflow (Additional file 2 supple Excel A2). A 1:1 million scale vector map of China’s administrative divisions, provided by the national basic geographic information system (http://bzdt.ch.mnr.gov.cn/index.jsp), was applied as the base map for analysis, and a sighting point map was generated by ArcGIS version 10.8 (http://www.esri.com/arcgis) (Fig. 1).

### Species distribution modeling

Environmental variables are crucial components influencing species distribution. Nineteen bioclimatic variables have been demonstrated to be the key variables for modeling potential species distributions [13]. Moreover, landscape features and socio-ecological changes such as forest protection and the reforestation of some areas are important environmental factors affecting human disease distribution [10, 11, 14]. Thus, four types of environmental variables that affect the distribution of *Echinococcus* spp. are recognized in this study: (1) climate variables, 19 bioclimatic factors from the WorldClim database [27], with a resolution of 30″, (2) topographic variables, including altitude, slope, and aspect, downloaded from the Geospatial Data Cloud Platform of the Computer Network Information Center of the Chinese Academy of Sciences (http://www.gscloud.cn/), (3) Vegetation variables, including vegetation coverage, land use type, and normalized vegetation index are obtained based on remote sensing interpretation data. The normalized difference vegetation index (NDVI) comes from the Chinese Academy of Sciences Geographical Sciences and Resource and Environmental Data Cloud Platform of the Resource Research Institute (http://www.resdc.cn/). (4) Water source variables, including the distance to the river and the distance to the lake, use the vector data of rivers and lakes in the ArcGIS software generated by the distance function. A pair of variables with Pearson’s correlation > |0.80| was removed and remained the most important factor for consideration in the final model according to permutation importance and ecological meaning. All remained variables were resampled in ArcGIS software, and the raster layer with a unified resolution of 1000 m is converted to ASCII format using the Asia North Albers Equal Area Conic projection coordinate system.

Future climate data (2070a) are downloaded (http://worldclim.org/) based on the BCC-CSM2-MR climate system model developed by the National Climate Center [28]. The model includes four emission scenarios proposed by the sixth International Coupled Models Comparison Program (CMIP6). WorldClim provides four shared socio-economic pathways (SPPs) for each model, corresponding to four CO_2_ emission scenarios. They are as follows: (1) SSP5–8.5: High obsessive-compulsive situation, Radial forcing stabilizes at 8.5 W.m^2^ in 2100. (2) SSP3–7.0: Moderate to the high obsessive-compulsive situation. Radial forcing stabilizes at 7.0 W.m^2^ in 2100. (3) SSP2–4.5 Moderate obsessive-compulsive situation: Radiative forcing stabilizes at 4.5 W.m^2^ in 2100. (4) SSP1–2.6 In the low obsessive-compulsive situation, radiative forcing stabilizes at 2.6 W.m^2^ in 2070 [29]. The Beijing Climate Center Climate System Model 2 Medium Resolution (BCC-CSM2-MR) of the National (Beijing) Climate Center Climate System Model and the Center National de Recherches Météorologiques (CNRM) and CNRM-CM6–1 jointly developed by the Center National de Recherches Météorologiques (CNRM) and the Cerfacs [30] were applied in this study.

MaxEnt software (Version 3.4.4, http://www.cs.princeton.edu/_schapire/maxent/) were applied to model the habitat suitability of *Echinococcus* spp. in Gansu Province (Additional file 3 supple Fig. A1). The algorithm does not require a comprehensive and systematic survey of the distribution of a certain species, but it can maximize the use of the limited biological survey data of certain species distribution in a certain area over the years [31–33]. For further analyses, the results of MaxEnt were imported into ArcGIS version 10.8, and five categories of potential future habitats were reclassified as follows: very high potential (> 0.8), high potential (0.6 ~ 0.8), good potential (0.4 ~ 0.6), moderate potential (0.2 ~ 0.4), and least potential (< 0.2) [34].

### Model evaluation

The area under the curve (AUC) of receiver operating characteristics [35] was applied to weight model performance. An AUC value lower than 0.7 indicates poor prediction accuracy, 0.7 to 0.9 indicates medium prediction accuracy and higher than 0.9 indicates high prediction accuracy of the predicted output of the model. Using random seeds, we repeat 15 times to calculate the average result [36].

### Data analysis

Microsoft Excel and SPSS 11.5 (version 19.0; SPSS Inc., Chicago, IL) were applied to compare the prevalence and analyzed the risk factors linked with the demographic information. *P* < 0.05 was examined as statistically significant.

## Results

### Clinical characteristics of echinococcosis in Gansu Province

A total of 626 hospitalized HE patients were identified between 2000 and 2020. Organ involvement in all hospitalizations was as follows: 89.14% (558 cases) in liver infection, 0.32% (2 cases) in lung infection, 8.14% (51 cases) in other organ infection, and 2% (12 cases) in multiple organ infection.

The mean age of the 626 HE patients (males: females = 1.04 (319 vs. 307)) was 48.32 ± 15.62 years (range 2–89), with the 40–60 age group being slightly more indicative (45.69%). Of the 626 hospitalizations, 430 (68.69%) registered a surgical intervention. The average hospitalization duration was 16.14 days. Among the surgical inpatients, 62% of HE cases remained in the hospital for 14 days, 23% of which were hospitalized for less than or equal to 10 days, with up to 4 months of hospital stay in complicated cases (ranging from 1 to 125 days).

**Fig. 2:**
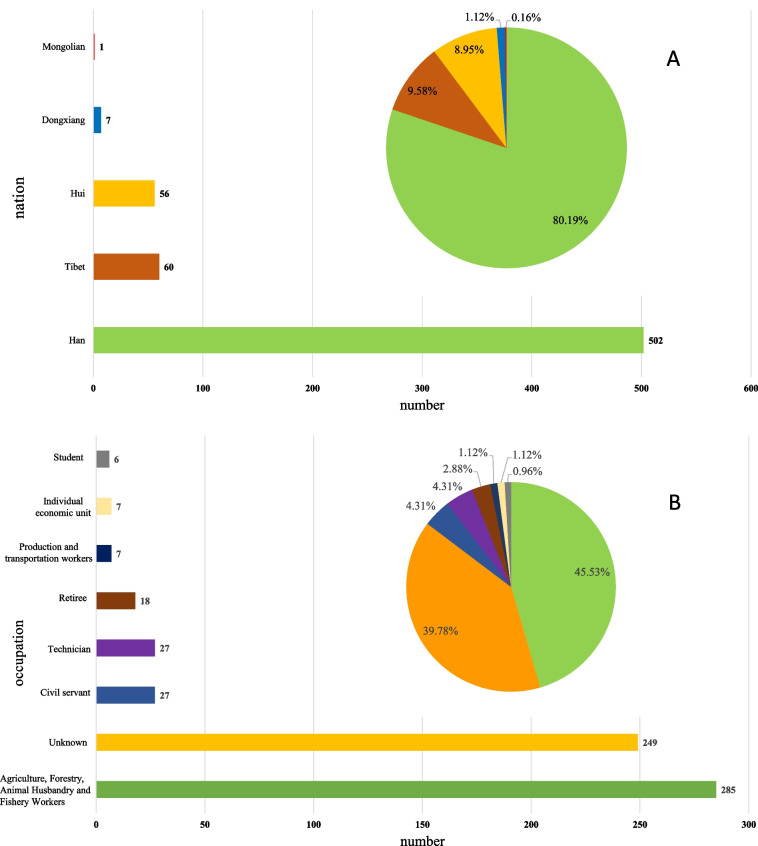
Ethnic (**A**) and occupational (**B**) distributions of 626 patients from 2000 to 2020 in Gansu Province, China

In the statistics of ethnic distribution, Han nationality accounted for the largest proportion (80.19%), followed by Tibetan (9.58%) and Hui nationality (8.95%, Fig. 2 A). Among the 626 patients, 285 patients were employed as agriculture, forestry, animal husbandry, and fishery workers. Twenty-five of the patients were government officials and civil servants, including two centers for disease control and prevention (CDC) staff, and 27 patients were professional technicians including 4 leather factory workers. Seven patients were engaged in transportation (Fig. 2 B). Of 626 cases, 158 patients were from Lanzhou city (25.24%).

### Spatial and temporal socio-economic cost and trends in Gansu Province

**Fig. 3:**
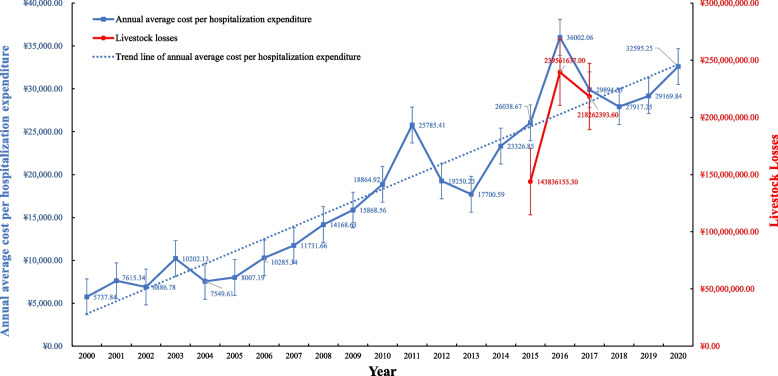
Annual average cost per hospitalization expenditure due to AE and CE from 2000 to 2020 and annual livestock losses due to CE from 2015 to 2017 in Gansu Province, China

The hospitalization median cost was ￥24,370.20 (35.6–22,8622) (Table 1). Western medicine accounted for the largest proportion of the total expenses (33.91%), the cost of the surgery itself (18.66%), and other components of the total expenses (22.28%) (Table 1). The general hospitalization expenses of echinococcosis patients increased with an annual growth rate of 8.95% from 2000 to 2020 (Fig. 3). The trend of livestock losses was increased but the highest loss was in 2016 (Fig. 3), which is similar to average cost of HE hospital expenses (Fig.3).

**Table 1:** Hospitalization Expenses for 523 Patients with HE from 2000 to 2020 in Gansu Province, China

Item	Average	Median	Total	Proportion
Blood transfusion fees	216.23	0.00	131,791.55	0.89%
Chinese Medicine fees	336.17	0.00	183,249.73	1.38%
Bed fees	408.11	345.00	215,324.00	1.68%
Nursing expenses	1164.61	306.00	627,358.86	4.79%
Treatment fees	1773.48	1393.68	966,679.99	7.29%
Examination fees	2215.57	1749.00	1,180,556.90	9.11%
Operation expenses	4538.67	3627.40	2,383,635.29	18.66%
Medicine fees	8245.75	6138.19	4,365,887.92	33.91%
Other expenses	5419.11	3699.78	2,871,046.50	22.28%
Aggregate	24,317.70	21,837.63	12,925,530.74	100.00%

**Fig. 4:**
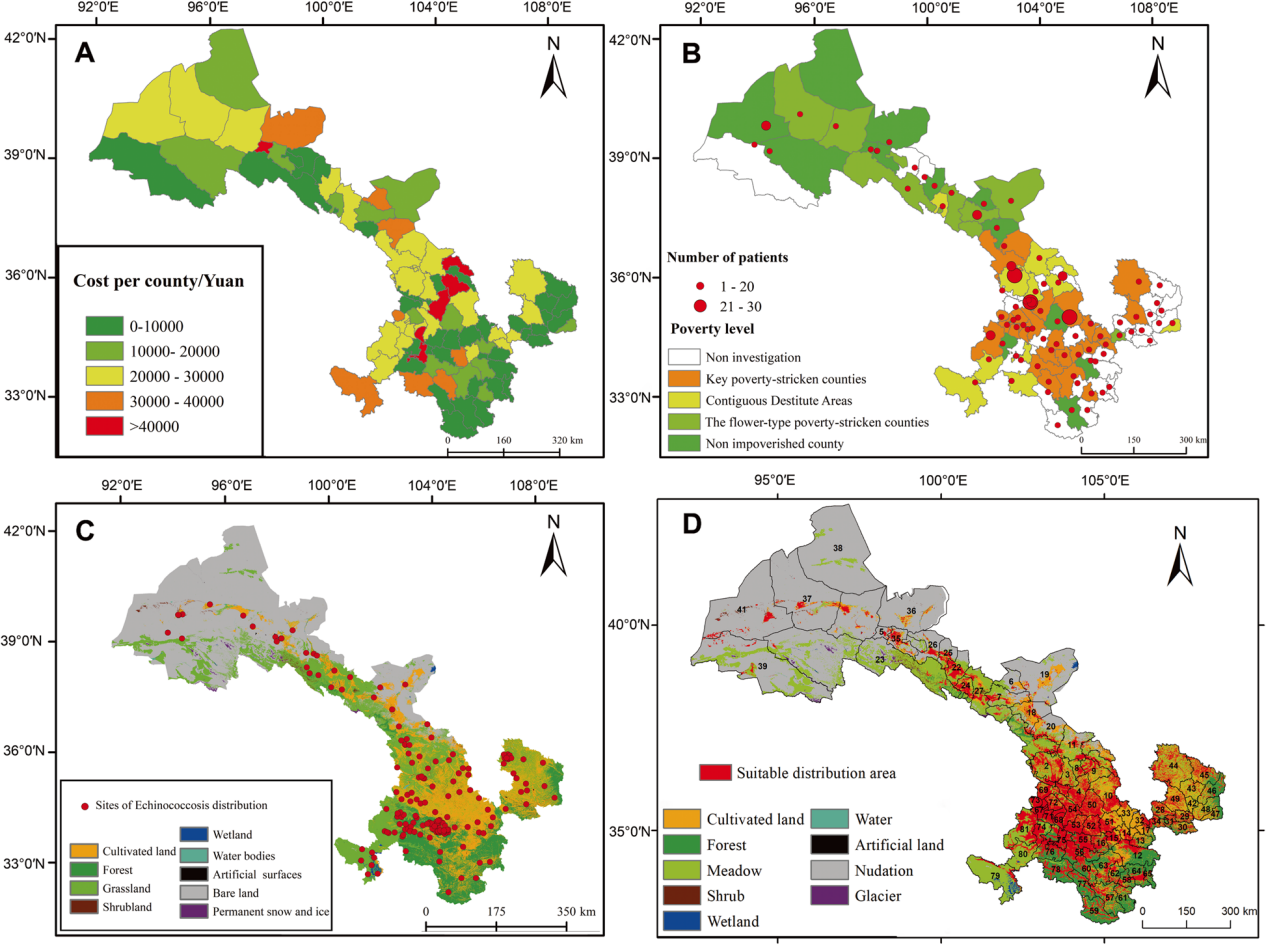
Information of Gansu Province. **A** Average expenditure per case in different Counties; **B** The occurrence records sites (see Additional file 1 supple Excel A1) in different poverty-stricken counties in Gansu Province (sizes of the red spots indicate the number of patients); **C** case locations (red spots) in different land types in Gansu Province from 2000 to 2022. **D** Results of the MaxEnt distribution model of *Echinococcus* spp. under different land types. Binary prediction of suitable environmental conditions using two different reclassification thresholds. The red distribution represents the suitable area using a threshold that equates of the threshold and original distributions. Numbers indicate different counties or districts (See Additional file 1 supple Excel A1). Note Map as created with ArcMap from ArcGIS 10.8. The land cover vector layers were acquired from the Department of Natural Resources in Gansu Province, China

The cost of treating a case of echinococcosis in different counties was significantly negative with their corresponding GDP (Fig. 4 A). Human cases are distributed in different poverty-stricken counties, and a higher hospitalization median cost occurs in the key poverty-stricken counties (Fig. 4 B) and 9 land types of Gansu province (Fig. 4 C).

### Contributions of environmental variables

Variable response curves show how each environmental variable affected the MaxEnt prediction, indicating how the logistic prediction changes with the alteration of each environmental variable. Three types of lands, i.e., “cultivated lands” (code 80), “waterbodies” (code 60), and “artificial surfaces” (code 10), were highly associated with the presence of *Echinococcus* spp. (Additional file 5 supple Fig. A2a). To attempt to reduce the influence of correlated variables on the analysis, one variable from each pair with correlation coefficients above than 0.8 was deleted. Variables that have been presented to be more important or meaningful were remained in the analysis. Of the 9 variables (Table 2) applied for modeling, the significant factors influencing the spatial distribution of *Echinococcus* spp. were land cover types (56.6% of variations), annual precipitation (21.2% of variations), and mean temperature of the Wettest Quarter (8.5% of variations) of the total variation. The variables with high permutation importance were precipitation in the warmest quarter (22.7%) and land cover types (20.4%). The additional information shows in more detail the respective response curves of the variables utilized in the MaxEnt model (Additional file 5 supple Fig. A2).

**Table 2:** Contribution of environmental variables in MaxEnt model for *Echinococcus* spp.

Abbreviation	Description	Unit	Permutation importance	Percent Contribution
altitude	Altitude	m	7.9	1.4175
bio_03	Isothermality (BIO2/BIO7) (* 100)	–	1.9	3.9365
bio_04	Temperature Seasonality (standard deviation *100)	°C	16.9	3.2874
bio_08	Mean Temperature of Wettest Quarter	°C	10.3	8.5282
bio_12	Annual Precipitation	mm	13.9	21.3446
bio_14	Precipitation of Driest Month	mm	2.7128	1.4221
bio_18	Precipitation of Warmest Quarter	mm	22.715	1.7305
bio_19	Precipitation of Coldest Quarter	mm	2.1019	1.1406
dis_river	The distance to a river	km	1.1917	0.5573
glc10	10 land cover types	–	20.4187	56.6353

### Suitable distribution area under current conditions

By using the MaxEnt model, the model predicted the potential distribution of *Echinococcus* spp. in Gansu Province, with a training AUC value of 0.929 and a test AUC value of 0.888, which indicates its high level of predictive performance (Additional file 5 supple Fig. A2b).

Using the reclassification tool of ArcMap from ArcGIS 10.8, based on the maximum training sensitivity plus specificity logistic threshold in the MaxEnt result as the threshold, the logical output results generated using MaxEnt software are expressed in terms of probability and range between zero and one. Based on the presence data, the model gave the currently suitable habitats of *Echinococcus* spp. under current climate conditions and land cover types (Fig. 4 D). Linxia Hui Autonomous Prefecture (Code number 66–73 in Fig. 4 D), Gannan Tibetan Autonomous Prefecture (Code number 74–81 in Fig. 4 D), Dingxi City (Code number 50–56 in Fig. 4 D), Pingliang City (Code number 28–34 in Fig. 4 D), Qingyang City (Code number 42–49 in Fig. 4 D), Lanzhou City (Code number 1 in Code number Fig. 4 D), Baiyin City (Code number 8–11 in Fig. 4 D), Tianshui City (Code number 41–47 in Fig. 4 D) and Longnan City (Code number 32–40 in Fig. 4 D) were found to be suitable habitat areas for *Echinococcus* spp. in Gansu Province.

### Future changes in potential species distribution

**Fig. 5:**
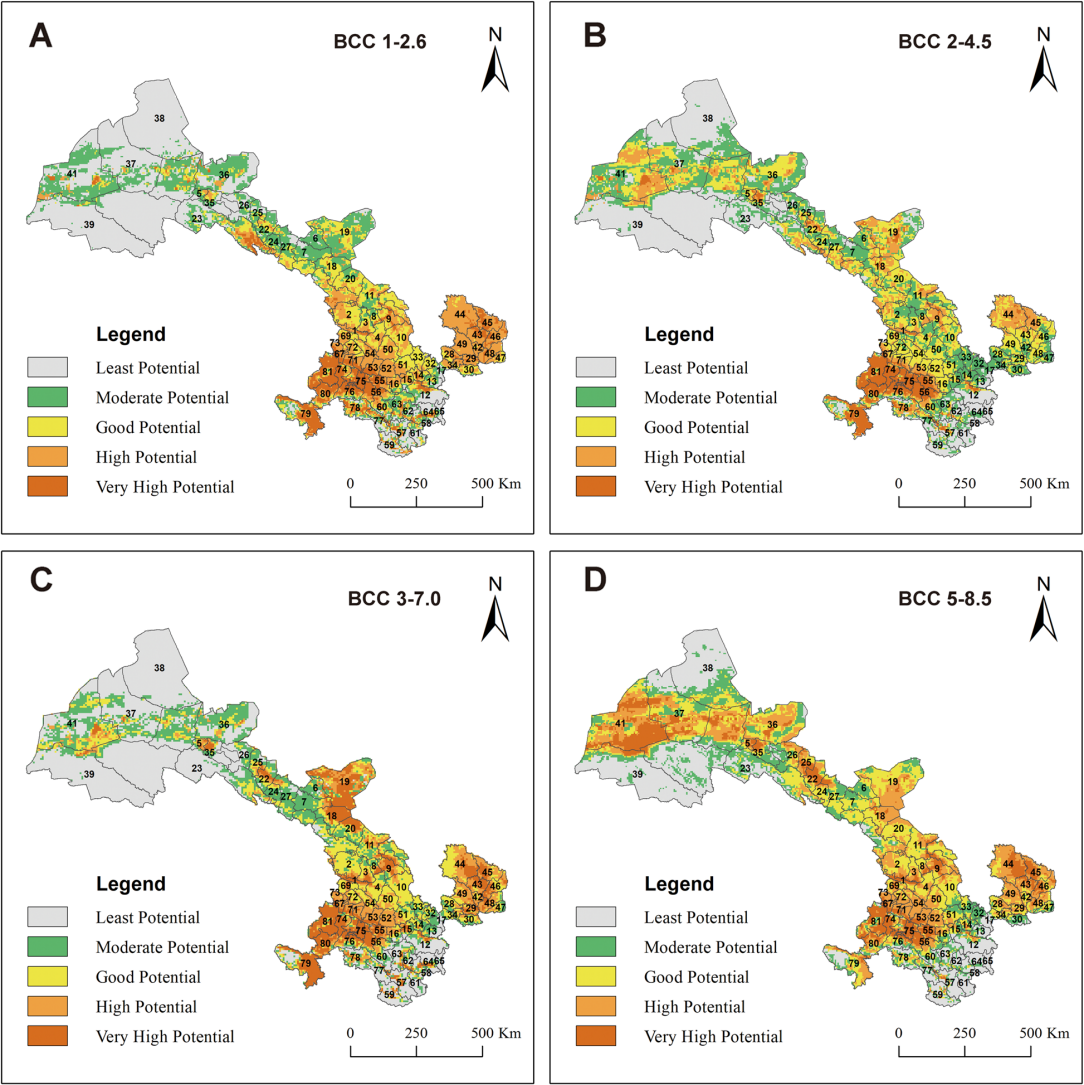
Future predictions (the 2070s) of *Echinococcus* spp. based on RCP8.5 under the conditions of the four shared economic pathway A) BCC 1-2.6, B) BCC 2-4.5, C) BCC 3-7.0 and D) BCC 5-8.5. Numbers indicate different counties or districts (See Additional file 1 supple Excel A1)

Under four emission scenarios, the MaxEnt model was applied to identify suitable habitat areas of *Echinococcus* spp. in Gansu Province. The relative changes in the future potential species distribution were estimated by the difference between current and future distribution maps, which revealed that the range expansion of *Echinococcus* spp. in Gansu Province was linked with the changing climate, socioeconomic and human behaviors (Fig. 5). Under the conditions of the four shared economic pathways (SSPs), the suitable distribution areas of *Echinococcus* spp. were expanded. The highly suitable area of *Echinococcus* spp. in Gansu Province changed to varying degrees, showing a significant increase in SSP5–8.5, while under the other three emission scenarios, the highly suitable area does not change significantly compared to that under current climate conditions. The highly suitable area of *Echinococcus* spp. increased significantly in the Linxia Hui Autonomous Prefecture (Code number 66–73 in Fig.5) and Gannan Tibetan Autonomous Prefecture (Code number 74–81 in Fig.5), with the most apparent increase under SSP5–8.5, and under the other three emission scenarios, the highly suitable area was not that significantly different. Among which has expanded the most obviously, Qingyang (Code number 42–49 in Fig.5), Wuwei (Code number 18–21 in Fig.5), and Jiuquan (Code number 35–41 in Fig.5) have also expanded significantly. Zhangye (Code number 22–27 in Fig.5), Jinchang (Code number 6–7 in Fig.5), Lanzhou (Code number 1–4 in Fig.5), Dingxi (Code number 50–56 in Fig.5), Linxia (Code number 66–73 in Fig.5), Tianshui (Code number 12–17 in Fig.5), and Longnan (Code number 57–65 in Fig.5) have not seen significant changes, but they are suitable habitats for *Echinococcus* spp. (Fig. 5).

## Discussion

To our knowledge, this is the first ecological niche model that has been fully developed and implemented for *Echinococcus* spp. in Gansu Province. The prevalence of *Echinococcus* spp. is not only a matter of ecological factors but also the result of the comprehensive effect of society, infrastructure, and human habitat changes in endemic or non-endemic areas [37]. Since 2005, the Chinese government has launched an echinococcosis control project for 217 counties in western China including Gansu Province [38]. Moreover, Gansu Province has become a new ecotourism hotspot, and tourists would expose to a high risk of endemic areas of *Echinococcus* spp. Raising travelers’ disease awareness and preventive health habits is an urgent agenda and is the most efficient-reaching action with a small investment.

### Case characteristics among patients with hydatid

Among the 626 patients, 89.14% had liver infection alone, which is consistent with previous research [39]. The proportion of lung infection is lower than in Herrador’s research (3.3%) [37]. The ratio of males vs females was 1.04, which is line with previous human case research [23, 26]. In this study, the prevalence of echinococcosis in the Han population was the highest (Fig. 2 B), which is line with human case research in Gansu Province [40] but inconsistent with the conclusion that the Tibetan population ratio was positively correlated with the prevalence of human CE [8]. This might be caused by different ethnic ratios in different research areas [40].

Most echinococcosis patients are engaged in agriculture and animal husbandry (46%, Fig. 2 A) in Gansu Province, which can be evidenced by previous research [25] but also health care workers involved. Hence, raising awareness of echinococcosis among health care workers [41] are urgent. Most cases are distributed in pastoral areas or mixed areas between pastoral areas and farms, but some research results show that the number of urban patients is increasing [2, 13], which can support an increase in the urban infection ratio (158/626 in our case vs 10/211) from previous research [23, 24]. The average length of hospital stay was 14 days, which is shorter than Chinese average hospital stays of 17.12 days in China [26].

The average cost of echinococcosis was ￥24,370.2, in Gansu Province, which is slightly higher than ￥ 21,201.85 [26]. The average cost of echinococcosis varied from year to year with an increasing trend (Fig. 3), which is consistent with the average hospitalization cost of Chinese patients with echinococcosis [26]. The rapid increasing of medical expenditures (Fig. 3), mostly caused by the frequent use of advanced medical technologies and over-prescription of drugs by health care providers [41]. We also found there were similar upward trend both livestock loss and average cost of HE hospital discharges (Fig. 3). It supports that Echinococcosis not only damages people’s health and life but also ruins the healthy growth of agriculture and animal husbandry economies [2]. They also shared the same highest cost and loss in 2016 (Fig. 3). They might be explained by their intermediate host of *Echinococcus spp* [2]*.* The average cost of echinococcosis differed significantly among regions (Fig. 4 A and B). The majority of echinococcosis patients live at a low-income level (< 4000￥/family/annum), and the number of patients is positively related to the level of poverty (Fig. 4 A and B), which indicates that a reduction in socio-economic disadvantage could contribute to risk of Echinococcosis [5, 41], and causes a heavy financial burden on sick families [4]. Thus, the prerequisite for effective disease control measures and appropriate policies and actions are essential from an economic point of view [3, 37]. It also could help to fulfill the Chinese government commission of eliminating poverty-impoverished areas [7].

### Epidemic characteristics of *Echinococcus* spp. in Gansu Province

Based on the distribution of the infected dogs and patients collected, we found that cultivated lands, water bodies, and artificial surfaces were more suitable land types for *Echinococcus* spp. (Table 2, Fig. 4 C), which supports that *Echinococcus* spp. distribution is related to land use and vegetation type [8]. Moreover, the Chinese government has restored the degraded ecological landscape to forestation [41]. Afforestation [15], and particular farming and fencing practices have been demonstrated to change the distribution of various species of *Echinococcus* spp. hosts. Landscape features and climate factors can predict human disease hotspots [11, 13]. Areas of artificial surfaces in Gansu Province during the last 2 decades have increased rapidly due to socio-economic development [32] since Gansu Province has restored arable land to nature. Future suitable areas of *Echinococcus* spp. are expanding under four scenarios (Fig. 5), which can be explained by predicted land cover in 2030 under the ecological protection scenario being more favorable [32].

Gannan Tibetan Autonomous Prefecture in southeast has a humid climate, low winter temperatures, and widely distributed livestock areas in Gansu Province [21], which keep it the most suitable habitats for *Echinococcus* spp. Suitable habitats of *Echinococcus* spp. is prominent toward the northwest in the 2070s (Fig.5 A-D). This might be caused by the Hexi areas showing an increasing annual rainy day and its potential epidemic risk [20]. Some areas possess a suitable environment for echinococcosis, but have been rarely reported, such as Longnan (Code number 57–65 in Fig. 4 D), Tianshui (Code number 12–17 in Fig. 4 D), Pingliang (Code number 28–34 in Fig. 4 D), and Zhangye (Code number 22–27 in Fig. 4 D). Identification of these areas may help in the development of policies for at-risk populations in geographically defined areas.

### Limitations to this study

There are several limitations to our study including the lack of future land cover types involved, which is inherent limitations of MaxEnt predicts. One is the assumption that the parasite niche will not evolve, and the other is the lack of wildlife host distribution data, but the host distribution will inevitably redistribute with climate change [18]. Nevertheless, it still has positive predictive significance. If regional social economic and land cover type in the future can be integrated into model, the resulting model will be more accurate [34]. This study did not separately analyze CE and AE, owing to a lack of information on the hospitalization record of echinococcosis types.

## Conclusions

This study provides the first attempt to determine the cost of hospitalization with their local GDP in Gansu Province. Spatial analyses of expenditure and the identification of non-endemic areas or risk for these parasites may help in the development of policies for at-risk populations in geographically defined areas. A better comprehension of the role of the environment in clarifying the transmission dynamics of *Echinococcus* spp. will help monitor improvements in human echinococcosis control strategies by allowing targeted allocation of resources. Raising health care workers and travelers’ disease awareness and preventive health habits is an urgent agenda.

## Supplementary Information

**Additional file 1.** Excel A 1. Geographical distribution of echococcosis

**Additional file 2.** Excel A 2. Geographical distribution of dogs with echococcosis from literatures

**Additional file 3.** Supple FigA1. Procedure of MaXent modeling

**Additional file 4.** Supple Table A1. Land types codes

**Additional file 5. **Supple FigA2. Modelling accuracy for predicting suitable habitats and Response curve results of MaxENT modelling of the environmental variables for potentially suitable habitat for *Echinococcus* spp.

## Data Availability

The Gansu Province geographic information data analyzed in the current study are available in the national basic geographic information system [http://bzdt.ch.mnr.gov.cn/index.jsp]. The climate variables analyzed in the current study are available in the WorldClim database, [http://worldclim.org/]. The topographic variables analyzed in the current study are available in the Geospatial Data Cloud Platform of the Computer Network Information Center of the Chinese Academy of Sciences, [http://www.gscloud.cn/]. The normalized difference vegetation index analyzed in the current study is available in the Chinese Academy of Sciences Geographical Sciences and Resource and Environmental Data Cloud Platform of the Resource Research Institute, [http://www.resdc.cn/]. The other datasets used and/or analyzed during the current study are available from the corresponding author on reasonable request.

## Abbreviations

GDP: Gross Domestic Product
AE: alveolar echinococcosis
CE: cystic echinococcosis
HE: human echinococcosis
ENMs: environmental niche models
GLC: global land cover
DRG: diagnosis-related groups
NVDI: normalized difference vegetation index
CMIP6: coupled model intercomparison project phase 6
SSPs: shared socio-economic pathways
BCC-CSM2-MR: Beijing climate center climate system model 2 medium resolution
CNRM: the center national de recherches météor ologiques
AUC: area under the curve
ROC: receiver operating characteristic
CDC: centers for disease control and prevention.
