# Supplementary figures and images for: How climate, landscape, and economic changes increase the exposure of Echinococcus Spp
Source: BMC Public Health. 2022 Dec 10;22:2315. doi: 10.1186/s12889-022-14803-4 (PMC9741777; doi:10.1186/s12889-022-14803-4)

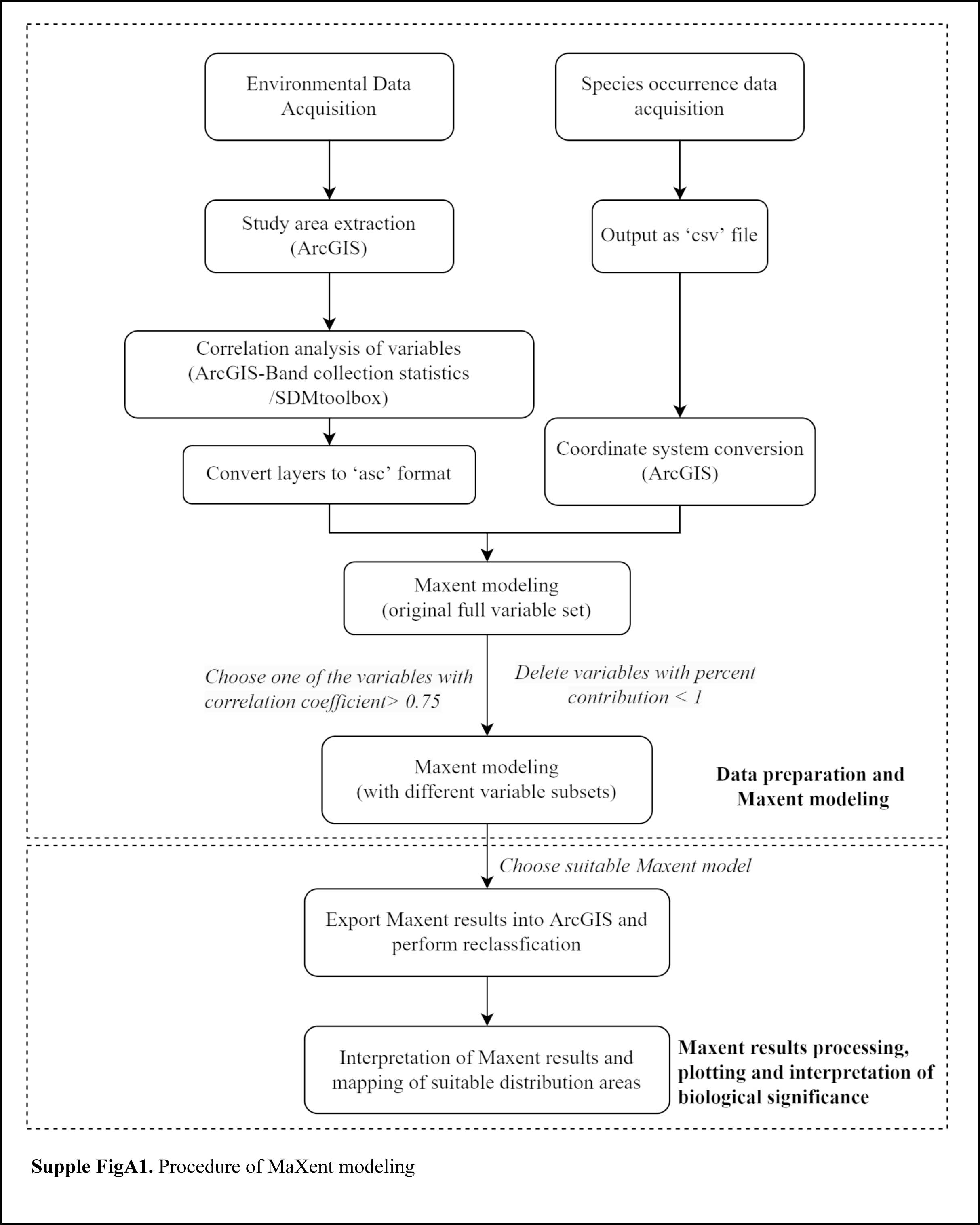

Supplement: Supplementary file 3 — Additional file 3. Supple FigA1. Procedure of MaXent modeling [file 12889_2022_14803_MOESM3_ESM.tif]

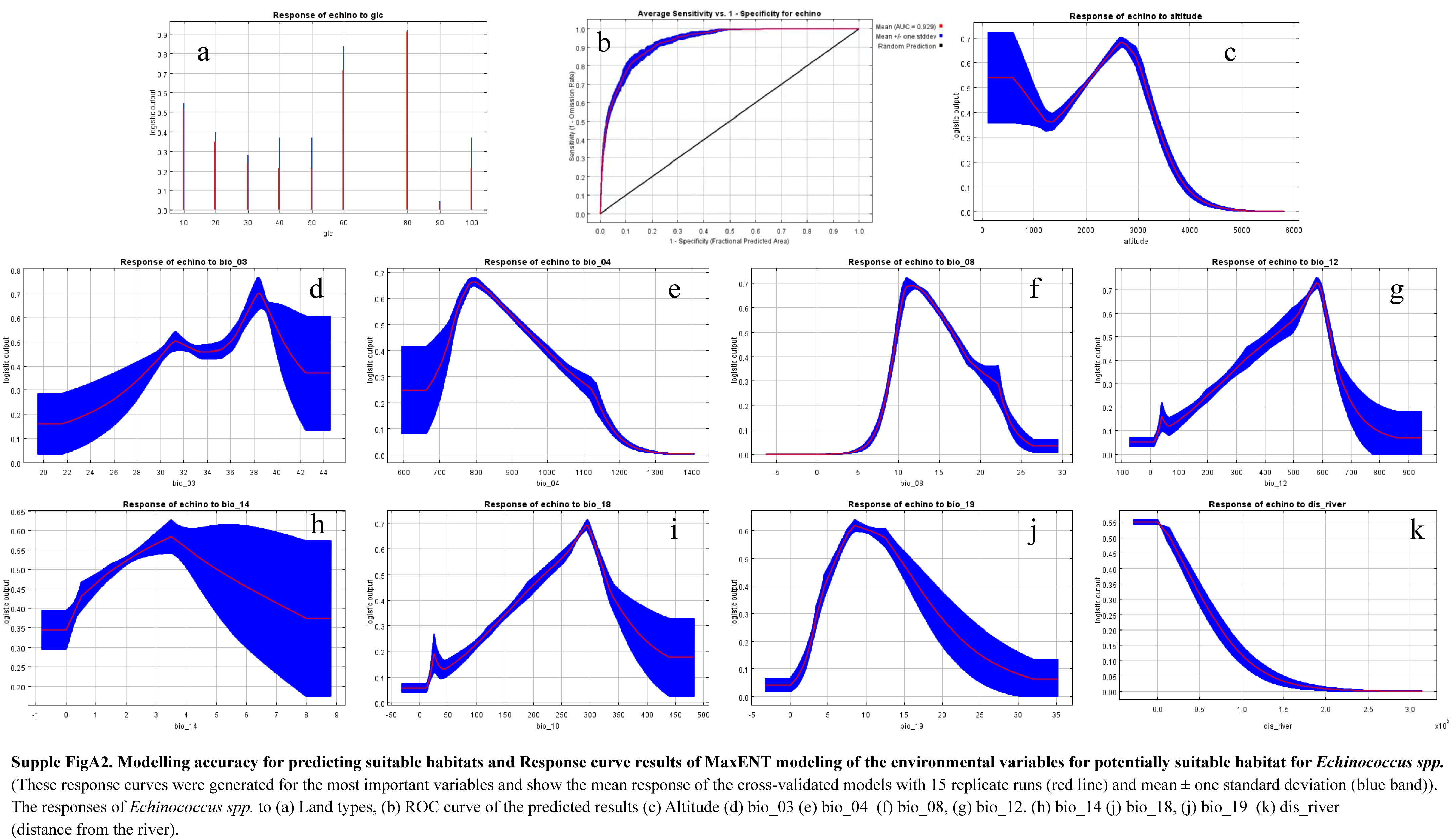

Supplement: Supplementary file 5 — Additional file 5. Supple FigA2. Modelling accuracy for predicting suitable habitats and Response curve results of MaxENT modelling of the environmental variables for potentially suitable habitat for Echinococcus spp. [file 12889_2022_14803_MOESM5_ESM.tif]
