# Supplementary material for: How climate, landscape, and economic changes increase the exposure of Echinococcus Spp
Source: BMC Public Health. 2022 Dec 10;22:2315. doi: 10.1186/s12889-022-14803-4 (PMC9741777; doi:10.1186/s12889-022-14803-4)
Supplement: Supplementary file 4 — Additional file 4. Supple Table A1. Land types codes [file 12889_2022_14803_MOESM4_ESM.docx]

**Supple Table A1 Land types codes**

| Land types | Code | Content |
| --- | --- | --- |
| Cultivated land | 10 | Land used for growing crops, including paddy fields, irrigated dry land, rain-fed dry land, vegetable land, pastureland, greenhouse land, land with fruit trees and other economic trees between mainly planted crops, as well as tea plantations, coffee plantations, and other shrubs for cash crops. |
| Woodland | 20 | Tree cover and canopy cover of more than 30% of the land, including deciduous broad-leaved forests, evergreen broad-leaved forests, deciduous coniferous forests, evergreen coniferous forests, mixed forests, as well as canopy cover of 10-30% of the open forest land. |
| Grassland | 30 | Land covered by natural herbaceous vegetation with a cover greater than 10%, including grasslands, meadows, savannas, desert grasslands, and urban artificial grasslands, etc. |
| Shrub | 40 | Land with shrub cover and more than 30% scrub cover, including mountain scrub, deciduous and evergreen scrub, and desert scrub with more than 10% cover in desert areas. |
| Wetlands | 50 | Land located in the border zone between land and water, with shallow standing water or excessively wet soil, mostly with boggy or wet plants growing. Includes inland bogs, lake bogs, river floodplain wetlands, forest/shrub wetlands, peat bogs, mangroves, salt marshes, etc. |
| Water | 60 | The area is covered by liquid water in the land area, including rivers, lakes, reservoirs, ponds, etc. |
| Tundra | 70 | Land covered by lichens, mosses, perennial hardy herbs, and shrubs vegetation in boreal and alpine environments, including scrub tundra, graminoid tundra, wet tundra, alpine tundra, bare ground tundra, etc. |
| Artificial surface | 80 | The surface is formed by artificial construction activities, including towns and other types of residential land, industrial and mining, transportation facilities, etc., excluding continuous green areas and water bodies within the construction land. |
| Bare ground | 90 | Natural cover land with less than 10% vegetation cover, including desert, sandy land, gravel land, bare rock and saline land, etc. |
| Glaciers and permanent snow | 100 | Land covered by permanent snow, glaciers, and ice caps, including permanent snow and glaciers in alpine areas, and polar ice caps. |
| Cultivated land | 10 | Land used for growing crops, including paddy fields, irrigated dry land, rain-fed dry land, vegetable land, pastureland, greenhouse land, land with fruit trees and other economic trees between mainly planted crops, as well as tea plantations, coffee plantations, and other shrubs for cash crops. |
